# Non-contrast cardiovascular magnetic resonance detection of myocardial fibrosis in Duchenne muscular dystrophy

**DOI:** 10.1186/s12968-021-00736-1

**Published:** 2021-04-29

**Authors:** Frank J. Raucci, Meng Xu, Kristen George-Durrett, Kimberly Crum, James C. Slaughter, David A. Parra, Larry W. Markham, Jonathan H. Soslow

**Affiliations:** 1 Thomas P Graham Division of Pediatric Cardiology, Department of Pediatrics, Vanderbilt University Medical Center, Nashville, TN USA; 2 Division of Pediatric Cardiology, Department of Pediatrics, Children’s Hospital of Richmond, Virginia Commonwealth University Medical Center, 1000 E. Broad St, Suite 5-344, Children’s Pavilion, Richmond, VA 23219 USA; 3 Department of Biostatistics, Vanderbilt University Medical Center, Nashville, TN USA; 4 Division of Cardiology, Department of Pediatrics, Riley Hospital for Children at Indiana University Health, Indianapolis, IN USA

**Keywords:** Duchene muscular dystrophy, Late gadolinium enhancement, Circumferential strain, Native T1, Cardiac fibrosis

## Abstract

**Background:**

Duchenne muscular dystrophy (DMD) leads to progressive cardiomyopathy. Detection of myocardial fibrosis with late gadolinium enhancement (LGE) by cardiovascular magnetic resonance (CMR) is critical for clinical management. Due to concerns of brain deposition of gadolinium, non-contrast methods for detecting and monitoring myocardial fibrosis would be beneficial.

**Objectives:**

We hypothesized that native T1 mapping and/or circumferential (ε_cc_) and longitudinal (ε_ls_) strain can detect myocardial fibrosis.

**Methods:**

156 CMRs with gadolinium were performed in 66 DMD boys and included: (1) left ventricular ejection fraction (LVEF), (2) LGE, (3) native T1 mapping and myocardial tagging (ε_cc-tag_ measured using harmonic phase analysis). LGE was graded as: (1) presence/absence by segment, slice, and globally; (2) global severity from 0 (no LGE) to 4 (severe); (3) percent LGE using full width half maximum (FWHM). ε_ls_ and ε_cc_ measured using feature tracking. Regression models to predict LGE included native T1 and either ε_cc-tag_ or ε_ls_ and ε_cc_ measured at each segment, slice, and globally.

**Results:**

Mean age and LVEF at first CMR were 14 years and 54%, respectively. Global ε_ls_ and ε_cc_ strongly predicted presence or absence of LGE (OR 2.6 [1.1, 6.0], *p* = 0.029, and OR 2.3 [1.0, 5.1], *p* = 0.049, respectively) while global native T1 did not. Global ε_cc_, ε_ls_, and native T1 predicted global severity score (OR 2.6 [1.4, 4.8], *p* = 0.002, OR 2.6 [1.4, 6.0], *p* = 0.002, and OR 1.8 [1.1, 3.1], *p* = 0.025, respectively). ε_ls_ correlated with change in LGE by severity score (n = 33, 3.8 [1.0, 14.2], *p* = 0.048) and ε_cc-tag_ correlated with change in percent LGE by FWHM (n = 34, OR 0.2 [0.1, 0.9], *p* = 0.01).

**Conclusions:**

Pre-contrast sequences predict presence and severity of LGE, with ε_ls_ and ε_cc_ being more predictive in most models, but there was not an observable advantage over using LVEF as a predictor. Change in LGE was predicted by ε_ls_ (global severity score) and ε_cc-tag_ (FWHM). While statistically significant, our results suggest these sequences are currently not a replacement for LGE and may only have utility in a very limited subset of DMD patients.

**Supplementary Information:**

The online version contains supplementary material available at 10.1186/s12968-021-00736-1.

## Introduction

Duchenne muscular dystrophy (DMD) leads to progressive skeletal and cardiac myopathy and affects up to 1 in 4700 live male births [1]. Disease progression is variable, but all boys will have cardiovascular manifestations by 18 years of age [2]. There is no cure for DMD. While cardiovascular therapeutic options are limited, early therapy with angiotensin converting enzyme inhibitors and/or aldosterone inhibitors has demonstrated delayed mortality [3, 4]. Some boys with DMD have earlier onset of cardiovascular disease or more rapid progression, requiring either earlier initiation or more rapid intensification of therapy, resulting in early heart failure and death.

Cardiovascular imaging is necessary to detect both early onset and rapid progression of cardiomyopathy. While transthoracic echocardiography has been the mainstay of cardiovascular evaluation in DMD, the field has increasingly shifted to cardiovascular magnetic resonance (CMR) [5]. This is partially because CMR provides superior image quality, a significant advantage in a patient population with challenging acoustic windows [6]. CMR also allows for assessment of fibrosis with late gadolinium enhancement (LGE), which can precede left ventricular (LV) dysfunction and which predicts morbidity and mortality in multiple disease processes, including DMD [7–11]. Clinical decision making is often based on LGE in boys with DMD. Tandon and colleagues have shown that LV ejection fraction (LVEF) declines once LGE is present but not before [11]. Thus, LGE can be an early sign of impending LV dysfunction. In addition, given the increased mortality associated with LGE, a more rapid progression of LGE in the setting of stable or mild changes in LVEF is treated with more aggressive medical therapy. The benefit of LGE is the information it provides in addition to LVEF, especially in a patient with stable ventricular function. However, LGE requires administration of gadolinium contrast agents. While these agents have an excellent safety profile in patients without renal disease, recent reports suggest that renal disease may be under-recognized in older DMD patients [12], coupled with growing concern over possible long-term brain deposition after repeated scans has raised concerns about long-term safety in children and young adults [8, 13].

Recently, mapping of the longitudinal time constant, T1, has gained popularity for myocardial tissue characterization. Native T1 maps can be performed without contrast administration, while extracellular volume (ECV) maps require a combination of native T1 and post-contrast T1 maps as well as a recent hematocrit [14–16]. Native T1 times are elevated in the presence of myocardial fibrosis, and correlates with LGE in other disease processes [17–20]. We previously demonstrated that native T1 is elevated in DMD patients compared with healthy subjects [21]. Myocardial strain has also been used to determine the presence or absence of LGE [22]. We hypothesized that native T1 and/or circumferential (ε_cc_) and longitudinal strain (ε_ls_) can be used as surrogates for LGE in DMD subjects, allowing for less frequent gadolinium contrast administration.

## Methods

### Patient selection

Subjects were drawn from prospective observational studies, all of which were approved by the Vanderbilt Institutional Review Board. All patients signed approved consents or assents. Inclusion criteria were the following: (1) DMD diagnosed phenotypically and confirmed with either genetic testing or muscle biopsy, (2) at least one CMR performed. Exclusion criteria were: (1) other genetic diagnosis in addition to DMD, (2) renal disease or other diagnosis precluding CMR with contrast, (3) no LGE imaging performed on CMR, (4) inadequate T1 map quality in all three imaging slices on all available CMRs. As the incidence of renal disease is low in the general pediatric and DMD populations, creatinine is not usually assessed unless there is a clinical concern. However, screening blood chemistries including creatinine are performed clinically, particularly for patients on angiotensin converting enzyme inhibitors or aldosterone inhibitors. Of the patients enrolled in our studies, there were a total of 8 CMRs where a patient did not receive contrast and so were removed from our analysis. Of those, one was because of anxiety in the scanner so the patient was removed early, 7 were in patients in whom no intravenous access could be obtained or the intravenous catheter infiltrated.

### CMR protocol

CMR was performed on a 1.5T CMR system (Avanto, Siemens Healthineers, Erlangen, Germany). Functional imaging was performed as previously described using balanced steady state free-precession (bSSFP) images in a short axis stack [23]. Intravenous Gd-DTPA contrast (gadopentate dimeglumine, Magnevist^®^, Bayer Healthcare Berlin, Germany or gadobutrol, Gadovist^®^, Bayer Healthcare) was administered through a peripheral intravenous line at a dose of 0.2 mmol/kg. LGE imaging was performed using: (1) single shot (bSSFP) and segmented (turboFLASH) inversion recovery with optimized inversion recovery to null the signal from the myocardium, as well as phase sensitive inversion recovery (PSIR) bSSFP with an inversion time of 300 ms.

Myocardial tagging was performed in the LV short axis at the base, papillary muscles, and apex using a segmented k-space fast gradient echo sequence with electrocardiographic (ECG) triggering. Grid tagging was performed with a spacing of 8 mm and 9–13 phases. Typical imaging parameters included: slice thickness 6–8 mm, field-of-view 340 mm × 340 mm, matrix size 256 × 192, and minimum echo time and repetition time. The sequences were breath-holds and parallel imaging with generalized autocalibrating partially parallel acquisition (GRAPPA) with an acceleration factor of two. Breath-held modified Look-Locker inversion recovery (MOLLI) sequences (investigative) were performed prior to contrast administration in the LV short axis at the base, mid-LV, and apex at the same slice locations as the tagging [24]. MOLLI sequences were motion-corrected, ECG-triggered images obtained in diastole with typical imaging parameters: non-selective inversion with a 35° flip angle, single shot bSSFP imaging, initial inversion time of 120 ms with 80 ms increments, field-of-view 340 × 272 mm^2^, matrix size 256 × 144, slice thickness 8 mm, voxel size 1.3 × 1.9 × 8.0 mm^3^, TR/TE 2.6 ms/1.1 ms, parallel imaging factor of 2. The matrix size was decreased to 192 × 128 for heart rates > 90 (approximate voxel size 1.8 × 2.1 × 8 mm^3^; 72% of studies were performed with this smaller matrix size). The pre-contrast MOLLI acquired 5 images after the first inversion with the equivalent of a 3 s pause followed by 3 images after the second inversion, or 5(3s)3 (the number of heartbeats used for recovery was varied depending on the average heart rate just prior to T1 mapping: 3 beats for heart rate of 60, 4 beats for heart rate of 80, 5 beats for heart rate of 100, and 6 beats for heart rate of 120) [25]. Motion correction as described by Xue, et al. was performed and a T1 map was generated on the scanner [26]. A goodness of fit map was also performed at the time of the scan to evaluate data quality.

### CMR post-processing

LV volume, mass, and function were calculated as previously described [27]. The presence or absence of LGE was qualitatively assessed by one reader (JS). Global severity score was calculated as described previously, with a range of 0 (no LGE) to 4 (severe LGE) [10, 28]. A second reader (FR) performed a blinded analysis of 30 CMRs to evaluate reproducibility of global severity score. Percent of scar was calculated with the full width half maximum (FWHM) method using QMass (Medis Medical Imaging Systems, Leiden, The Netherlands) on the PSIR images. Analysis of myocardial tagged images was performed as previously described using harmonic phase (HARP) methodology (Myocardial Solutions, Morrisville, North Carolina, USA) [29]. In brief, a contour or mesh was drawn over the tagged image at peak systole by outlining the epicardium and endocardium. The superior right ventricular (RV) insertion was identified manually. The contours were performed by the same reader (KGD) with verification of each contour by a second reader (JHS) with more than 7 years of experience using the software. The software then automatically calculated the ε_cc-tag_ values for each segment (16 segment model) and slice (base, mid-LV, and apex). Our prior work has demonstrated excellent reproducibility for harmonic phase magnetic resonance (HARP) analysis [6].

Qstrain (Medis Medical Imaging) was used to calculate feature tracking circumferential and longitudinal strain (ε_cc_ and ε_ls_, respectively). For ε_cc_, cine images in the LV short axis at the base, mid-LV, and apex were chosen and for ε_ls_, cine images in the long axis 4-chamber, 3-chamber, and 2-chamber views were chosen. Endocardial borders were traced on each image at end diastole and end systole. Images were inspected closely to confirm adequate tracking and segments that did not track were removed from analysis; more than one segment in each slice with poor tracking resulted in the removal of that CMR from analysis. Global and segmental circumferential and longitudinal strain were calculated automatically by the software.

Using Qmaps (Medis Medical Imaging), epicardial and endocardial borders were manually drawn on native T1 maps within the LV mesocardium. The superior RV insertion was identified and automatically divided into segments using the American Heart Association standard 16 segment model [30]. Regions of interest were carefully traced to avoid partial volume averaging with blood-pool or epicardial fat. Based on the T1 mapping consensus statement, areas of LGE were not excluded as these areas were felt to be the most focal areas in a continuum of diffuse extracellular matrix expansion [31].

**Fig. 1:**
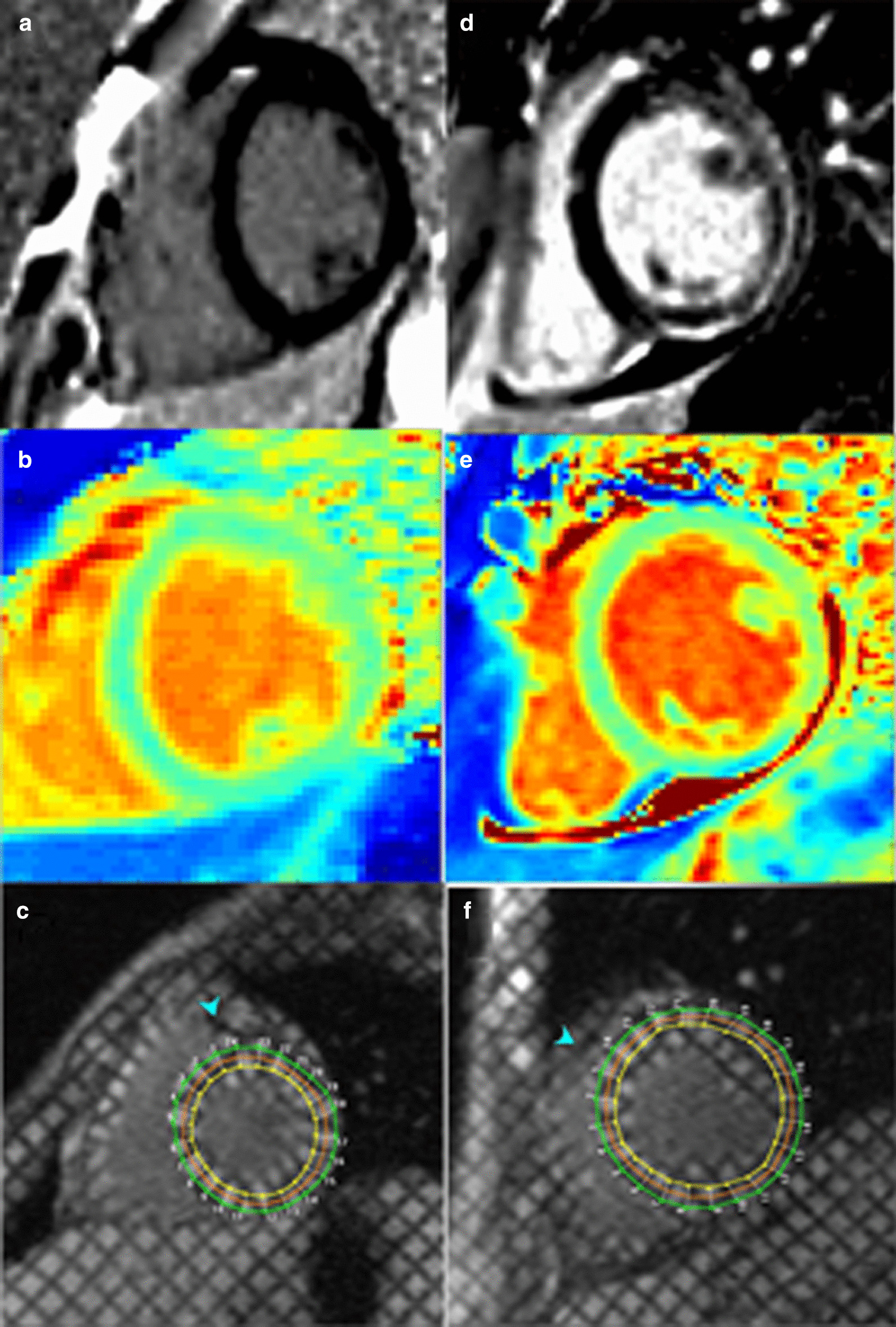
Representative image analysis for T1 and tagging. Representative late gadolinium enhancement (LGE) images (**a**, **d**), pre-contrast native T1 maps (**b**, **e**), and myocardial tagging (**c**, **f**) for patients without (**a**–**c**) and with (**d**–**f**) LGE

**Fig. 2:**
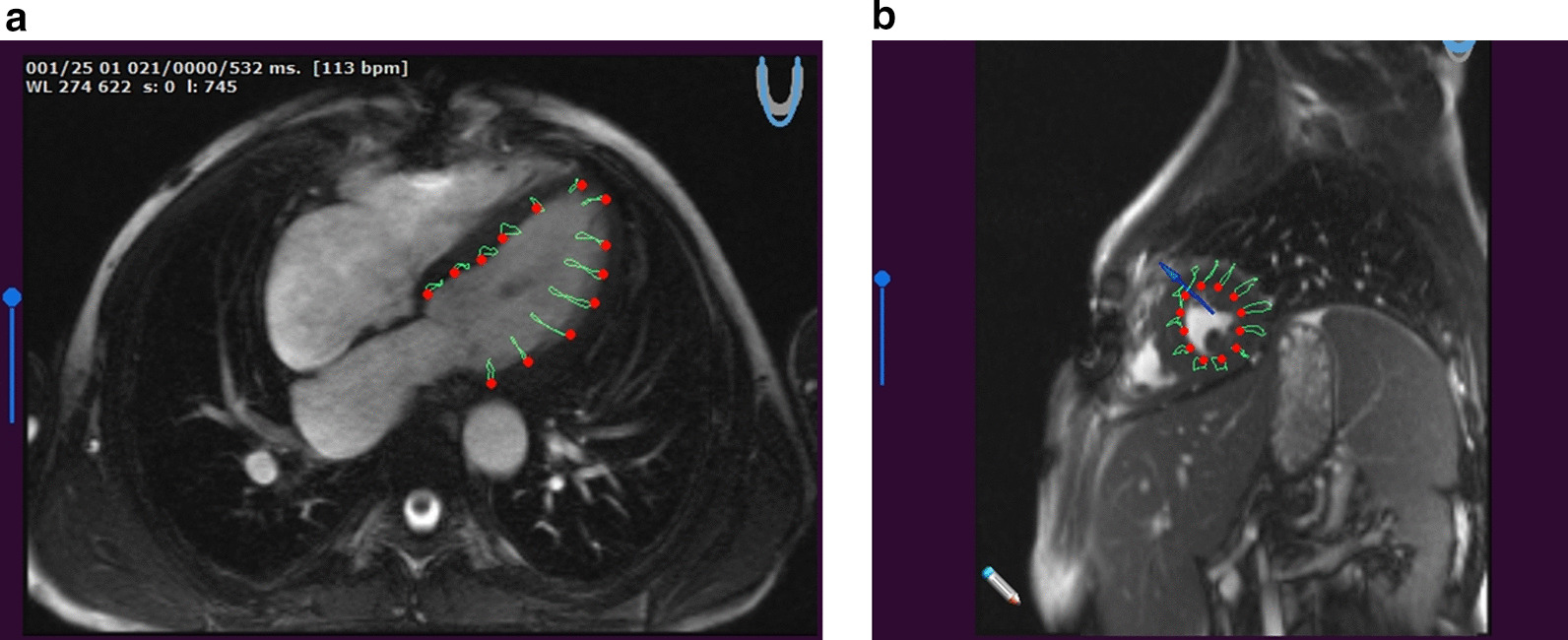
Representative image analysis for feature tracking. Examples of four-chamber view with tracking (**a**) and short axis for circumferential strain calculation (**b**)

In patients with adequate maps at the base and mid-LV slices, global myocardial T1 was calculated. Apical slices were not used to minimize errors from partial volume averaging. Imaging artifact was not contoured. For native T1 maps, segments were not included in the analysis if the bounds of the myocardium could not be distinguished from surrounding tissue and blood pool or if image registration was inadequate in those segments. The total number of excluded segments was 112 out of 2018, with 27/750 from the base, 23/864 from the mid-LV, and 62/404 from the apex. The mean myocardial wall thickness of the baseline CMRs was 6.7 ± 1.3 mm at the septum and 6.0 ± 0.9 mm at the free wall. Figure 1 demonstrates representative LGE, native T1, tagged images, and Fig. 2 shows an example of feature tracking analysis.

### Statistical analysis

Continuous data are presented as a mean and standard deviation. A missing data summary analysis was performed (Additional file 1: Figure S1). Reproducibility of global severity score was assessed using intraclass correlation coefficient and a weighted kappa. All-cause mortality at 5 years from first CMR in patients with and without LGE was evaluated using Cox regression analysis. Logistic, ordinal logistic, and linear regression models were used for binary outcomes, non-normally distributed continuous outcomes and change of the continuous outcomes respectively using Huber-White sandwich estimator to account for repeated measurements with ε_ls_, ε_cc_, and T1 mapping pre-specified as predictors and adjusting for age (and baseline measurements in the change model) as covariates.

Logistic, ordinal, and linear regression models were used to examine longitudinal progression. Progression was defined as: newly developed presence of LGE (logistic regression); any worsening of LGE global severity score (ordinal regression); any worsening of FWHM (linear regression). It should be noted that inclusion of LVEF in these models was considered; however, the decision was made to exclude LVEF and indexed LV end-systolic volume (LVESV) due to significant multicollinearity with strain parameters. We have included examples of univariate models for LVEF and indexed LVESV in Additional file 2: Figure S2). Analyses were performed with R version 3.5.2 (R Foundation for Statistical Computing, Vienna, Austria). Study data were collected and managed using REDCap (Research Electronic Data Capture) electronic data capture tools hosted at Vanderbilt [32].

## Results

### Demographics and outcomes

**Fig. 3:**
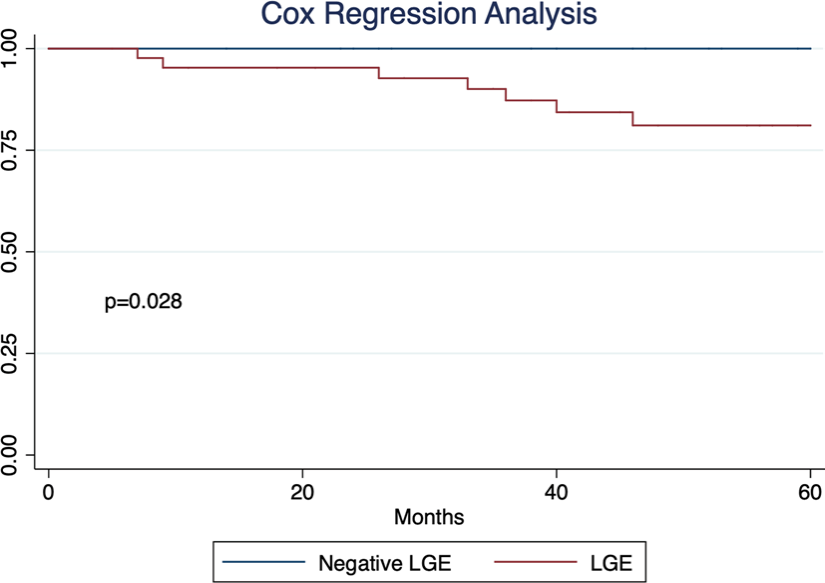
LGE Effect on Duchenne’s muscular dystrophy (DMD) survival. Cox regression survival analysis for our DMD cohort showed significant difference in all-cause mortality in patients with and without LGE

A total of 156 CMRs were analyzed from 66 enrolled patients (mean age and LVEF at first CMR were 14 years and 54.3%, respectively). The average total scan time was 40 min. At the time of the CMR, there were 33 patients on corticosteroids and 45 patients on cardiac medications. Seventeen patients were ambulatory. Forty-four patients (70%) were positive for LGE, with 12 patients having global severity score (GSS) = 1, 12 with GSS = 2, 13 with GSS = 3, and 7 with GSS = 4 (Table 1). Reproducibility analysis for GSS demonstrated an intraclass correlation coefficient of 0.99 (p < 0.001) and a weighted kappa of 0.97 (p < 0.001). Patients with LGE had significantly higher all-cause mortality (Fig. 3).

**Table 1:** Demographics at the time of their first cardiovascular magnetic resonance scan

	Mean ± SD or N (%)
Age (years)	14 ± 5
Height (m)	1.46 ± 0.18 (n = 65)
Weight (kg)	50.1 ± 17.8
BSA (m^2^)	1.41 ± 0.32
Ambulatory	17(26%)
Medications at baseline CMR	
Corticosteroid	33 (50%)
Angiotensin converting enzyme inhibitor	33 (50%)
Beta blocker	24 (36%)
Aldosterone inhibitor	4 (6%)
Angiotensin receptor blocker	6(9%)
LVEF (%)	54.3 ± 9.7
LVEDV (mL) LVEDVI (mL/m^2^)	93 ± 42 67 ± 20 (n = 65)
LVESV (mL) LVESVI (mL/m^2^)	45 ± 33 32 ± 17 (n = 65)
LV mass (g) Indexed LV mass (g/m^2^)	71 ± 27 49 ± 13 (n = 65)
Global native T1 (ms)	1048 ± 44 (n = 54)
Global ε_cc_ (%)	− 14.1 ± 3.5 (n = 64)
Presence of late gadolinium enhancement (LGE)	44 (70%)
Full width half maximum quantification of LGE (%)	21.3 ± 18.4 (n = 60)
GSS = 0	19 (30%)
GSS = 1	12 (19%)
GSS = 2	12 (19%)
GSS = 3	13 (21%)
GSS = 4	7 (11%)

N = 66 unless otherwise indicated

*BSA* body surface area, *GSS* global severity score, *LGE* late gadolinium enhancement, *LV* left ventricular, *LVEDV* left ventricular end-diastolic volume, *LVEDVI* left ventricular end-diastolic volume index, *LVEF* left ventricular ejection fraction, *LVESV* left ventricular end-systolic volume, *LVESVI* left ventricular end-systolic volume index

### Native T1 and strain predict the presence/absence and extent of LGE at a single time point

**Fig. 4:**
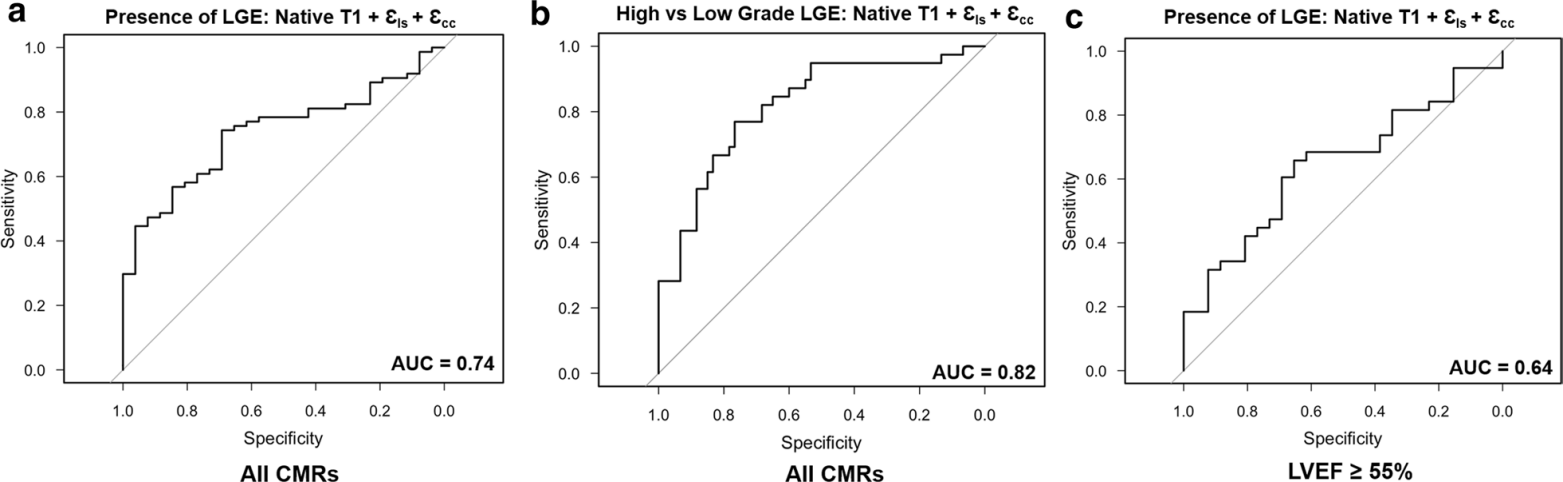
Receiver-operator curves for noncontrast variables. Plots for combined model with native T1, Ɛ_cc_, and ε_ls_ with modest areas under the curve (AUC) for prediction of presence/absence of LGE for all CMRs (**a**) and in the subset with normal left ventricular ejection fraction (LVEF) (**c**). The best performance was for predicting high grade [global severity score (GSS) ≥ 3] vs low grade (GSS ≤ 2) for all CMRs (**b**)

A model including global ε_ls_, ε_cc_, and native T1 predicted the presence or absence of LGE, though native T1 failed to reach significance (Table 2), with a correlation statistic (C) = 0.74 and r^2^ = 0.21, n = 95 (Fig. 4a). ε_cc_, ε_ls_, and native T1 all correlated with global severity score (Table 2), however only global ε_ls_ correlated with percent LGE by FWHM (Table 2). There was not sufficient power to estimate specific cutoffs for the individual variables so combined prediction models for predicting presence/absence of LGE and high grade (GSS ≥ 3) vs low grade (GSS ≤ 2) were created (Fig. 4a, b). Thresholds were determined for the best sensitivity and specificity (with a minimum of 40% in the other) as well as for the optimal sensitivity/specificity combination (Table 3). Sensitivity, specificity, and thresholds for univariate LVEF analysis is included as a reference.

**Table 2:** Models for prediction of presence and severity of late gadolinium enhancement (LGE)

Slice	Factor	Odds ratio and 95% CI		*p* value
Presence/absence of LGE				
Base (*n* = 105)	Native T1	2.1 [1.2, 3.8]		***0.012***
	ε_cc_	2.6 [1.1, 6.1]		***0.034***
	ε_ls_	2.3 [1.1, 4.8]		***0.032***
Mid (*n* = 133)	Native T1	1.2 [0.8, 1.7]		0.42
	ε_cc_	2.4 [1.2, 4.7]		***0.009***
	ε_ls_	2.3 [1.4, 3.7]		***0.001***
Apex (*n* = 106)	Native T1	1.4 [0.7, 2.8]		0.30
	ε_cc_	1.9 [0.9, 4.1]		0.12
	ε_ls_	1.0 [0.4, 2.6]		0.93
Global (*n* = 121)	Native T1	1.5 [0.8, 2.6]		0.17
	ε_cc_	2.3 [1.0, 5.1]		***0.049***
	ε_ls_	2.6 [1.1, 6.0]		***0.029***
Global severity score				
Base (*n* = 104)	Native T1	2.2 [ 1.1, 4.6]	***0.026***	
	ε_cc_	3.3 [1.2, 3.3]	***< 0.001***	
	ε_ls_	2.0 [1.9, 5.7]	***0.006***	
Mid (*n* = 132)	Native T1	1.3 [ 1.0, 1.9]	0.085	
	ε_cc_	2.8 [1.7, 4.6]	***< 0.001***	
	ε_ls_	2.1 [1.5, 3.1]	***< 0.001***	
Apex (*n* = 105)	Native T1	0.9 [ 0.6, 1.5]	0.25	
	ε_cc_	1.8 [0.9, 3.4]	0.077	
	ε_ls_	1.8 [0.9, 3.2]	0.085	
Global (*n* = 120)	Native T1	1.8 [ 1.1, 3.1]	***0.025***	
	ε_cc_	2.6 [1.4, 4.8]	***0.002***	
	ε_ls_	2.6 [1.4, 4.7]	***0.002***	
FWHM				
Base (*n* = 103)	Native T1	1.4 [0.9, 2.2]	0.19	
	ε_cc_	2.0 [1.1, 3.5]	***0.017***	
	ε_ls_	1.5 [1.0, 2.2]	***0.038***	
Mid (*n* = 114)	Native T1	1.1 [0.8, 1.6]	0.58	
	ε_cc_	2.2 [1.3, 3.5]	***0.002***	
	ε_ls_	1.7 [1.1, 2.5]	***0.014***	
Apex (*n* = 103)	Native T1	1.0 [0.6, 1.5]	0.85	
	ε_cc_	1.3 [0.8, 2.3]	0.26	
	ε_ls_	0.9 [0.5, 1.6]	0.70	
Global (*n* = 119)	Native T1	1.3 [0.8, 2.2]	0.28	
	ε_cc_	1.4 [0.9, 2.2]	0.19	
	ε_ls_	2.0 [1.2, 3.5]	***0.01***	

Bold italic values are significant for *p* ≤ 0.05

*FWHM* full width half maximum

**Table 3:** Threshold performance for models incorporating native T1, ɛ_ls_, and ɛ_cc_

Outcome condition	Model fit equation	AUC	R^2^	Threshold	Sensitivity (%)	Specificity (%)	+ LR	− LR
Presence/absence LGE	0.0096 * (native T1) + 0.2263 * (ɛ_ls_) + 0.538 * (ɛ_cc_)	0.74	0.21	0.473	81.1	42.3	1.4	0.45
				0.839	74.3	69.2	2.4	0.37
				1.512	44.6	96.2	11.7	0.58
	LVEF (univariate)	0.81	–	56%	56.8	99.9	568	0.43
High (GSS 3–4) vs low (GSS 0–2) grade LGE	0.013 * (native T1) + 0.32 * (ɛ_ls_) + 0.059 * (ɛ_cc_)	0.82	0.38	− 1.297	94.9	53.3	2.0	0.10
				− 0.559	76.9	76.7	3.3	0.30
				0.672	43.6	93.3	6.5	0.60
	LVEF (univariate)	0.87	–	50%	63.8	93.8	10.3	0.39

*GSS* global severity score, *AUC* area under the curve, *+ LR* positive likelihood ration, − *LR* negative likelihood ratio, *LVEF* left ventricular ejection fraction

**Fig. 5:**
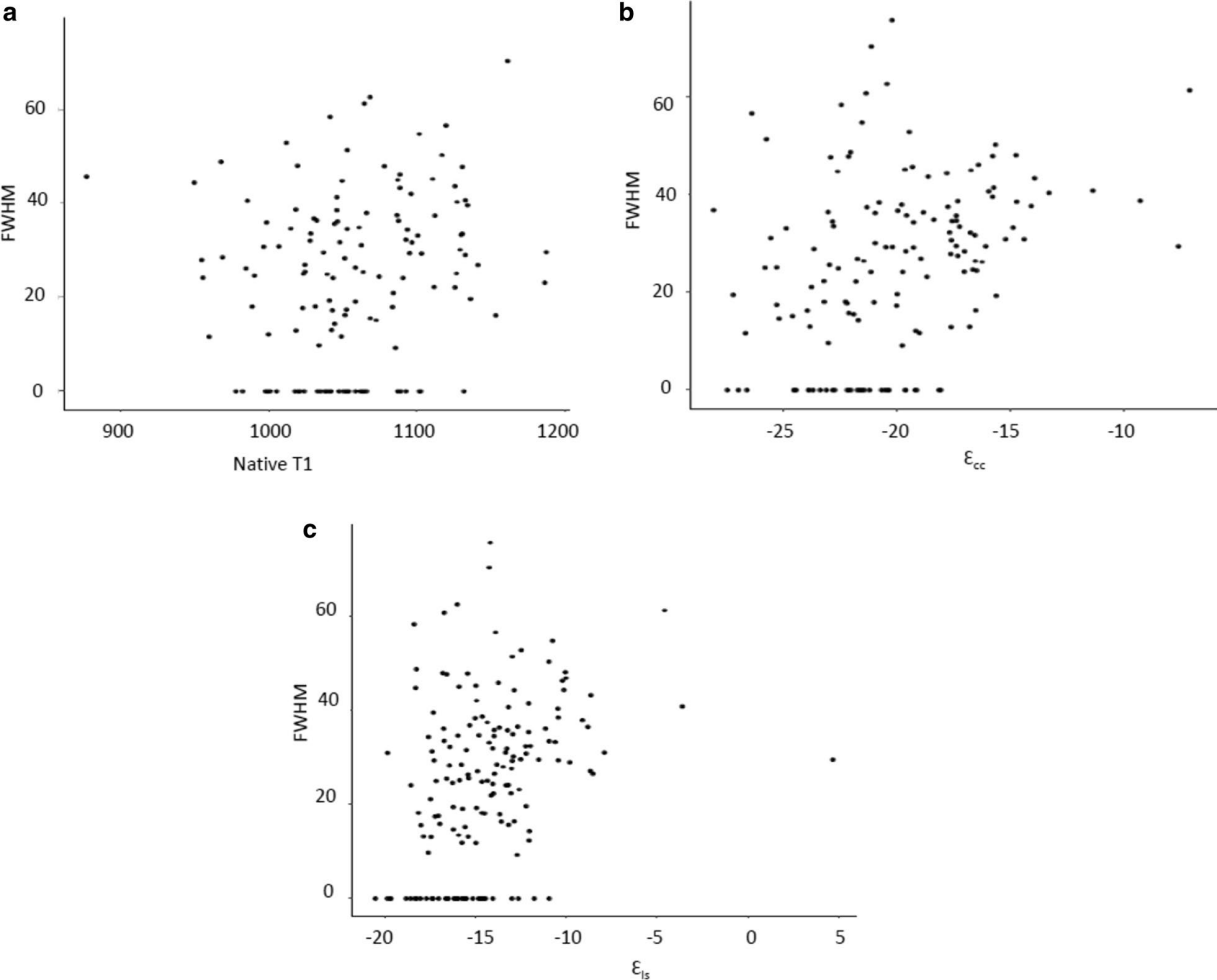
Distribution of noncontrast variables as a function of full width half maximum (FWHM). Scatter plots for native T1 (**a**), Ɛ_cc_ (**b**), and ε_ls_ (**c**) vs FWHM

For tagged analysis, global ε_cc-tag_ was predictive of presence/absence of LGE (n = 132, OR 3.3 [1.7, 6.4], *p* < 0.001) and GSS (n = 131, OR 2.6 [2.2, 6.4], *p* < 0.001) while native T1 was predictive of GSS but not presence/absence of LGE (Additional file 3: Table S1). Scatter plots for the distribution of FWHM values versus global native T1 (A), global ε_cc_ (B), and global ε_ls_ (C) are shown in Fig. 5.

A subset analysis was performed to evaluate presence of LGE in subjects with a normal LVEF. Given the smaller number of CMRs (total n = 95, 55 with LGE), the model performed poorly, with a C = 0.64 and r^2^ = 0.057. Figure 4c shows receiver operator curve for the model with ε_cc_, ε_ls_, and native T1 for DMD patients with LVEF ≥ 55% (n = 95).

Models evaluating percent LGE by slice (base, mid, and apex) as predicted by strain and T1 at each slice were also evaluated. Basal and mid-LV ε_ls_, ε_cc_, and ε_cc-tag_ were predictive of presence or absence of LGE, global severity score, and to a lesser extent FWHM (Table 2, Additional file 3: Table S1). Native T1 was only predictive in the basal slices.

For individual segments, ε_cc_ and/or ε_ls_ predicted presence/absence of LGE in almost all segments assessed except for the basal anterior, mid inferolateral, and mid anterolateral segments (Table 4). Native T1 failed to significantly predict presence/absence of LGE in most segments assessed and neither native T1, ε_ls_, nor ε_cc_ were reliably predictive of segmental percent LGE (Additional file 4: Table S2). For tagged data, ε_cc-tag_ was predictive of LGE presence and extent by FWHM for mid-LV segments (Additional file 4: Table S2).

**Table 4:** Models for prediction of presence/absence LGE by segment

Segment	Factor	LGE presence/absence	*p* value
		Odds ratio and 95% CI	
Basal anterior	Native T1	1.2 [0.9, 1.6]	0.33
	ε_cc_	1.3 [0.9, 1.9]	0.21
	ε_ls_	1.1 [0.7, 1.9]	0.72
Basal anteroseptal	Native T1	1.1 [0.7, 1.6]	0.73
	ε_cc_	1.4 [0.9, 2.2]	0.17
	ε_ls_	1.9 [1.2, 3.0]	***0.01***
Basal inferoseptal	Native T1	1.5 [1.1, 2.1]	***0.01***
	ε_cc_	3.6 [2.4, 5.5]	***< 0.001***
	ε_ls_	1.9 [1.2, 3.0]	***0.01***
Basal inferior	Native T1	2.0 [1.2, 3.4]	***0.01***
	ε_cc_	2.7 [1.7, 4.1]	***< 0.001***
	ε_ls_	1.1 [0.7, 1.6]	0.77
Basal inferolateral	Native T1	2.0 [1.4, 3.3]	***0.003***
	ε_cc_	2.9 [1.8, 4.7]	***< 0.001***
	ε_ls_	1.4 [0.9, 2.1]	0.21
Basal anterolateral	Native T1	1.3 [0.8, 2.1]	*0.25*
	ε_cc_	4.6 [2.8, 7.7]	***< 0.001***
	ε_ls_	1.8 [1.0, 3.3]	***0.042***
Mid anterior	Native T1	0.9 [0.6, 1.2]	0.38
	ε_cc_	2.0 [1.3, 3.2]	***0.001***
	ε_ls_	1.2 [0.8 1.7]	0.38
Mid anteroseptal	Native T1	1.0 [0.7, 1.5]	0.99
	ε_cc_	1.5 [0.9, 2.6]	0.15
	ε_ls_	1.9 [1.3, 2.8]	***< 0.001***
Mid inferoseptal	Native T1	1.3 [0.8, 2.2]	0.26
	ε_cc_	3.2 [1.9, 5.6]	***< 0.001***
	ε_ls_	1.6 [1.0, 2.4]	***0.04***
Mid inferior	Native T1	0.9 [0.5, 1.5]	0.59
	ε_cc_	2.3 [1.3, 4.1]	***0.004***
	ε_ls_	1.4 [1.0, 1.9]	***0.05***
Mid inferolateral	Native T1	1.4 [0.8, 2.2]	0.25
	ε_cc_	1.2 [0.5, 2.9]	0.67
	ε_ls_	2.8 [1.9, 4.3]	***< 0.001***
Mid anterolateral	Native T1	0.9 [06, 1.4]	0.59
	ε_cc_	1.2 [0.5, 3.2]	0.70
	ε_ls_	1.3 [0.9, 2.0]	0.17

Bold italic values are significant for *p* ≤ 0.05

### Longitudinal assessment of LGE using native T1 and strain in individual patients

**Fig. 6:**
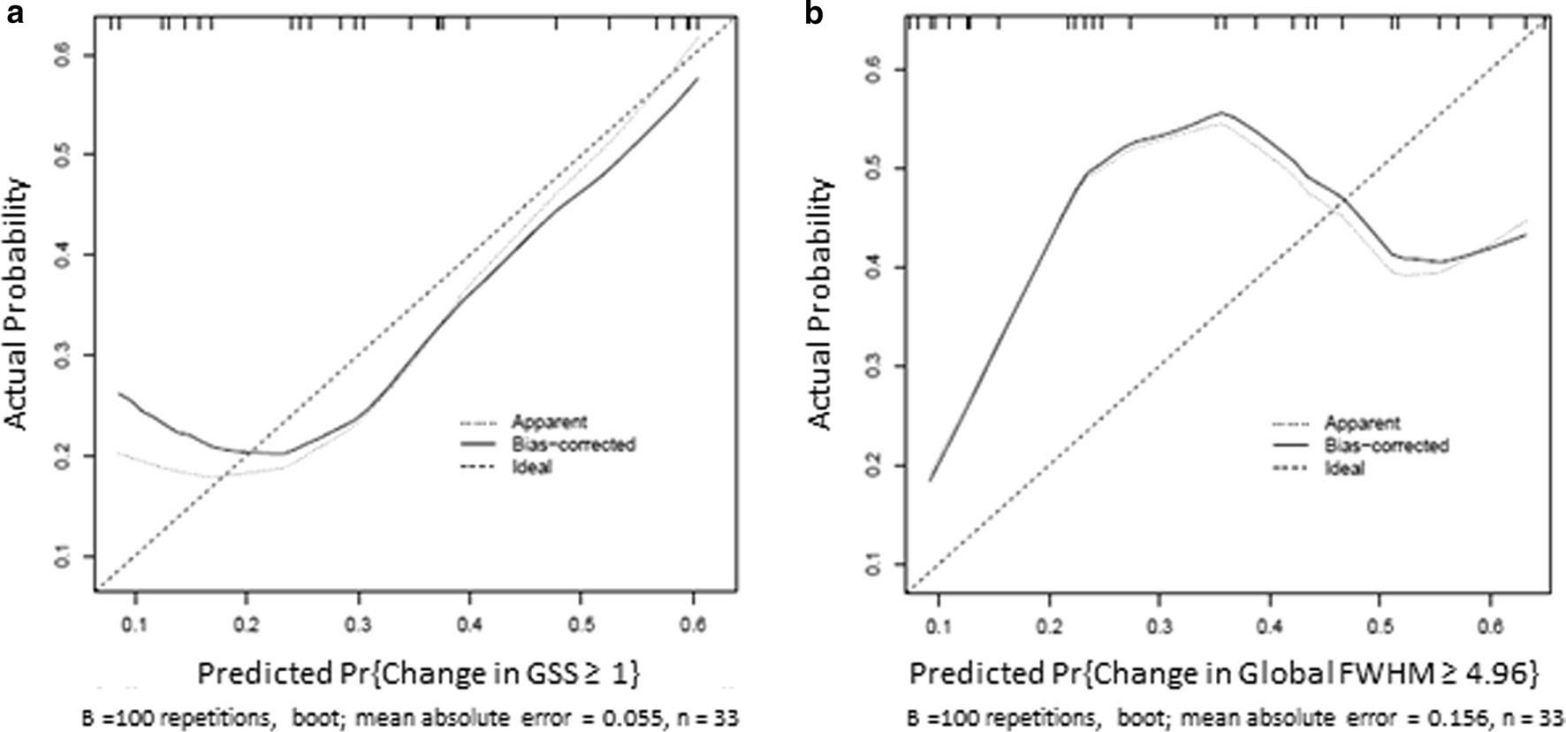
Predicted vs actual probability of change in LGE. Model performance for global severity score ≥ 1 (**a**) and change in FWHM (**b**)

Fifty-five patients had more than one CMR and were included in the longitudinal analysis. Mean changes in follow-up time, LVEF, and CMR parameters between studies are shown in Table 5. The mean interval between the first and second CMR was 423 ± 200 days and the mean interval between the first and third CMR was 748 ± 344 days. Ordinal logistic regression modeling for change in GSS using native T1, ε_cc_, ε_ls_, and initial global LGE demonstrated poor predictive power at the base with a C = 0.663 and r^2^ = 0.158 (Table 6). The model performed modestly better for native T1, Ɛ_cc_, ε_ls_, and initial global LGE at the mid slice (C = 0.784 and r^2^ = 0.327) and globally (C = 0.752 and r^2^ = 0.288, Fig. 6a). Linear regression modeling for change in percent LGE using FWHM (Table 6) demonstrated modestly worse correlation statistics for models using the same parameters at the base (C = 0.616, r^2^ = 0.145), mid (C = 0.642, r^2^ = 0.234), and globally (C = 0.659, r^2^ = 0.267) (Fig. 6b). Change in FWHM was predicted by change in ε_cc-tag_ at base (C = 0.585, r^2^ = 0.233), mid (C = 0.617, r^2^ = 0.235), and globally (C = 0.636, r^2^ = 0.355, Table 6). There was not sufficient power to detect incremental change in GSS, although this trended toward significance (*p* = 0.084, data not shown).

**Table 5:** Characteristics of longitudinal assessment

	N	Mean ± SD or N (%)
CMR 1 to CMR 2		
Interval (days)	55	423 ± 200
∆LVEF_1–2_ (%)	55	− 2.6 ± 4.7
Base ∆Ɛcc_1–2_	54	0.7 ± 3.0
Mid ∆Ɛcc_1–2_	55	0.7 ± 3.3
Apex ∆Ɛcc_1–2_	52	0.6 ± 3.4
∆FWHM_1–2_	37	4.3 ± 14.2
∆LGE_1–2_	53	
− 1		4 (8%)
0		31 (58%)
1		15 (28%)
2		2 (4%)
3		1 (2%)
CMR 2 to CMR 3		
Interval (days)	35	376 ± 26
∆LVEF_2–3_ (%)	35	− 2.4 ± 5.5
Base ∆Ɛcc_2–3_	34	0.4 ± 3.1
Mid ∆Ɛcc_2–3_	34	0.8 ± 3.1
Apex ∆Ɛcc_2–3_	32	0.1 ± 3.8
∆FWHM_2–3_	27	3.2 ± 16.3
∆LGE_2–3_	35	
− 1		4 (11%)
0		17 (49%)
1		11 (31%)
2		3 (9%)
CMR 1 to CMR 3		
Interval (days)	35	748 ± 344
∆LVEF_1–3_ (%)	35	− 5.1 ± 5.8
Base ∆Ɛcc_1–3_	35	1.2 ± 3.2
Mid ∆Ɛcc_1–3_	34	1.3 ± 3.4
Apex ∆Ɛcc_1–3_	34	0.7 ± 4.1
∆FWHM_1–3_	23	9.0 ± 16.6
∆LGE_1–3_	35	
− 1		2 (6%)
0		15 (43%)
1		13 (37%)
2		2 (6%)
3		3 (9%)

**Table 6:** Models predicting change in LGE severity

Slice	Factor	Odds ratio and 95% CI	*p* value
Change in global severity score (feature tracking)			
Base (*n* = 27)	Native T1	1.2 [ 0.3, 5.0]	0.78
	ε_cc_	2.2 [0.6, 7.7]	0.21
	ε_ls_	1.8 [0.8, 3.9]	0.15
	LGE at CMR1	0.6 [0.2, 1.9]	0.37
Mid (*n* = 30)	Native T1	1.2 [0.5, 2.7]	0.64
	ε_cc_	0.3 [0.1, 1.1]	0.07
	ε_ls_	3.0 [1.3, 6.7]	***0.008***
	LGE at CMR1	0.3 [0.1, 1.5]	0.13
Global (*n* = 33)	Native T1	1.4 [0.6, 3.3]	0.44
	ε_cc_	0.5 [0.2, 1.2]	0.14
	ε_ls_	3.8 [ 1.0, 14.2]	***0.048***
	LGE at CMR1	0.5 [0.1, 1.7]	0.25
Change in global severity score (myocardial tagging)			
Base (*n* = 42)	Native T1	1.3 [ 0.5, 3.2]	0.59
	ε_cc-tag_	0.8 [0.4, 1.8]	0.56
	LGE at CMR1	0.4 [0.1, 1.3]	0.13
Mid (*n* = 44)	Native T1	1.5 [0.9, 2.4]	0.11
	ε_cc-tag_	0.6 [0.3, 1.2]	0.15
	LGE at CMR1	0.5 [0.2, 1.6]	0.22
Global (*n* = 38)	Native T1	1.5 [0.7, 2.9]	0.27
	ε_cc-tag_	0.5 [0.2, 1.2]	0.12
	LGE at CMR1	0.3 [0.1, 1.2]	0.10
Change in FWHM (feature tracking)			
Base (*n* = 27)	Native T1	1.2 [0.3, 4.8]	0.83
	ε_cc_	1.5 [0.4, 6.8]	0.57
	ε_ls_	1.9 [0.7, 4.9]	0.19
	LGE at CMR1	0.6 [0.1, 3.7]	0.57
Mid (*n* = 30)	Native T1	0.8 [0.4, 1.6]	0.55
	ε_cc_	0.4 [0.1, 1.9]	0.25
	ε_ls_	1.5 [0.5, 4.6]	0.48
	LGE at CMR1	0.1 [0, 1.5]	0.10
Global (*n* = 33)	Native T1	0.9 [0.4, 2.2]	0.84
	ε_cc_	0.7 [0.2, 2.7]	0.61
	ε_ls_	2.0 [0.9, 4.5]	0.10
	LGE at CMR1	0.1 [0, 0.8]	***0.033***
Change in FWHM (myocardial tagging)			
Base (*n* = 36)	Native T1	1.7 [ 0.7, 4.1]	0.26
	ε_cc-tag_	0.6 [0.5, 0.9]	***0.003***
	LGE at CMR1	0.3 [0.1, 1.6]	0.16
Mid (*n* = 40)	Native T1	1.6 [0.7, 3.8]	0.32
	ε_cc-tag_	0.4 [0.1, 0.9]	***0.036***
	LGE at CMR1	0.2 [0.1, 1.1]	*0.058*
Global (*n* = 34)	Native T1	1.8 [0.7, 4.6]	0.21
	ε_cc-tag_	0.2 [0.1, 0.7]	***0.01***
	LGE at CMR1	0.2 [0.03, 1.1]	*0.075*

Bold italic values are significant for *p* ≤ 0.05

Changes in individual segments were also modeled. A model including change in ε_cc_, ε_ls_, and native T1 mapping had moderate predictive power for segmental LGE changes by severity score but was less accurate for FWHM (Table 7 and Additional file 5: Table S3). Similar results were seen with tagging (Additional file 6: Table S4).

**Table 7:** Models for prediction of change in LGE severity by segment

Segment	Factor	Change in severity score	*p *value
		Odds ratio and 95% CI	
Basal anterior	Native T1	1.6 [1.0, 2.7]	0.06
	ε_cc_	1.4 [0.8, 2.4]	0.30
	ε_ls_	2.9 [1.2, 7.1]	***0.02***
Basal anteroseptal	Native T1	3.5 [1.6, 7.6]	***0.002***
	ε_cc_	1.1 [0.4, 3.1]	0.87
	ε_ls_	0.4 [0.3, 0.8]	***0.012***
Basal inferoseptal	Native T1	1.4 [1.0, 2.1]	0.07
	ε_cc_	1.1 [0.3, 1.0]	0.55
	ε_ls_	0.5 [0.3, 1.0]	***0.048***
Basal inferior	Native T1	0.5 [0.2, 1.5]	0.23
	ε_cc_	0.4 [0.1, 1.3]	0.11
	ε_ls_	2.2 [1.0, 4.9]	***0.042***
Basal inferolateral	Native T1	2.2 [0.4, 11.6]	0.35
	ε_cc_	1.1 [0.8, 1.5]	0.11
	ε_ls_	5.6 [1.1, 29.4]	***0.042***
Basal anterolateral	Native T1	1.5 [1.0, 2.2]	0.47
	ε_cc_	0.6 [0.2, 2.2]	0.49
	ε_ls_	4.0 [0.8, 20.1]	0.10
Mid anterior	Native T1	1.5 [0.6, 3.8]	0.41
	ε_cc_	1.6 [0.6, 4.2]	0.37
	ε_ls_	0.8 [0.5, 1.5]	0.52
Mid anteroseptal	Native T1	1.0 [0.9, 1.0]	0.07
	ε_cc_	0.4 [0.2, 1.1]	0.06
	ε_ls_	1.5 [0.3, 7.8]	0.63
Mid inferoseptal	Native T1	2.3 [0.6, 9.1]	0.22
	ε_cc_	1.1 [0.7, 1.8]	0.55
	ε_ls_	0.3 [0.1, 3.0]	0.31
Mid inferior	Native T1	0.4 [0.2, 0.8]	***0.006***
	ε_cc_	0.1 [0, 0.5]	***0.007***
	ε_ls_	2.4 [0.8, 7.1]	0.13
Mid inferolateral	Native T1	2.9 [1.1, 8.3]	***0.042***
	ε_cc_	0.5 [0.1, 2.0]	0.33
	ε_ls_	1.8 [0.4, 9.2]	0.48
Mid anterolateral	Native T1	1.2 [0.7, 2.1]	0.46
	ε_cc_	0.9 [0.7, 1.1]	0.34
	ε_ls_	1.7 [0.4, 7.5]	0.45

Bold italic values are significant for *p* ≤ 0.05

## Discussion

This study presents the largest cohort of CMRs in DMD patients evaluating ε_cc_, ε_ls_, and native T1. The primary findings of this study are: (1) modeling using pre-contrast sequences modestly predicts presence and severity of LGE for individual CMRs; (2) strain appears more predictive than native T1 in most models; (3) strain, but not native T1 or initial global LGE, predicts change in severity of LGE estimated by GSS (for feature tracking) and FWHM (for myocardial tagging); (4) Non-contrast sequences are not a substitute for LGE imaging in DMD. While strain was statistically predictive of most outcome variables, the performance of strain was only modest, and native T1 was not predictive in most models. The performance of most non-contrast models evaluated did not meet thresholds for routine clinical use, emphasizing the importance of LGE in the DMD population.

Native T1 mapping has shown promise as a tool for detecting myocardial fibrosis in dilated cardiomyopathy as assessed by LGE and histologically [33]. Given the regionality of fibrosis observed in DMD cardiomyopathy, the utility of this sequence may be in selected segments rather than as a global predictor. Apical slices are susceptible to errors from partial volume averaging and also have less fibrosis than basal and mid-LV slices in DMD boys. Global values were thus calculated excluding apical slices. Recently, Olivieri and colleagues found that native T1 and ECV in the lateral wall were increased in DMD boys with LGE compared to those without LGE, though there was no significant difference in the septum [34]. They also found that the saturation recovery single shot acquisition (SASHA) technique was better at discriminating between disease states (control, DMD no LGE, and DMD with LGE) than MOLLI; however, modeling suggested that MOLLI could also distinguish between DMD boys with and without LGE. Studies suggest that native T1 values of myocardium and, to a lesser extent blood pool, remain relatively stable in healthy adults [35] and those with arrhythmias [36]. However, systematic longitudinal assessment of native T1 reproducibility has not been well characterized in dilated cardiomyopathies. There are many factors that may have affected the association of T1 maps with LGE in the DMD patient population. Fatty infiltration has been well-described in skeletal muscle and may be present with progressive myocardial disease. This could lower native T1– thus, T1 values may depend on the ratio of fibrosis to fat in LGE. Indeed, a recent study suggested native T1 decreases with worsening LGE in DMD boys, which the authors hypothesized was secondary to fatty infiltration [37]. DMD boys also may have lower image quality as they age, as many DMD boys have progressive respiratory weakness, limiting ability for adequate breath-holding. This limitation should be corrected in most cases with the motion correction and we excluded patients with poor map quality. Positioning and image quality may lead to scan-to-scan variation in native T1 maps. This may lead to inaccurate estimation of the change in native T1. Finally, while LGE demonstrates replacement fibrosis, native T1 is increased with both replacement and diffuse fibrosis; in addition, native T1 detects abnormalities in both myocytes and the extracellular matrix. Taken together, these factors may contribute to the poor performance of our models when using native T1 as one of the predictors for global severity as assessed by LGE.

By contrast, strain is a more functional assessment and areas demonstrating abnormal strain patterns secondary to fibrotic changes would be expected to progress as the pathological process progresses. Good correlation with ε_cc_ and myocardial fibrosis has been previously demonstrated based on histopathology and CMR assessment with LGE and ECV in adult patients with aortic stenosis [38] and hypertrophic cardiomyopathies [39]. Our data suggest that this holds true for dilated cardiomyopathy in DMD boys, with strain predicting both change in presence/absence and change in LGE extent in most of our models. Of note, while strain predicts change in extent of LGE, model performance is suboptimal (Fig. 6). Further, univariate analysis demonstrates that LVEF is at least as good as our multivariate models with native T1 and strain in predicting presence or absence of LGE in DMD patients with normal function (Additional file 1: Figure S1). While LVEF alone predicts the presence of LGE with high specificity (although less robust sensitivity), this is not as useful in clinical practice as the population of interest is DMD patients with normal or stable LVEF and progression of LGE. Interestingly, our model performs better in terms of predictive power for all DMD boys compared to the subset with normal LVEF (≥ 55%) and our models have better negative likelihood ratios than LVEF alone (Table 3). This is consistent with studies in adult populations with chronic heart failure, where addition of strain to LVEF had modest but significant improvements in AUCs [40]. This suggests a very limited role for strain, where one could consider using it only in those DMD boys who have an absolute contraindication to gadolinium or in whom intravenous access cannot be obtained. Given the suboptimal results of most models, the authors cannot recommend routine use of non-contrast CMR in place of LGE in patients without a contraindication to gadolinium. Despite the safety concerns associated with contrast, DMD subjects will likely continue to require conservative administration of gadolinium contrast for appropriate clinical management.

It is important to note the segmental distribution of the predictive models using native T1 and strain. Unlike many other forms of cardiomyopathy, the fibrosis seen in DMD dilated cardiomyopathy tends to be patchy and found in the subepicardial region, often in one or more segments of the lateral free wall. Segmental differences in native T1 and strain are more likely to be predictive in areas where LGE is usually found in DMD patients. Thus, there may be some benefit to monitoring for changes in segmental parameters even in patients with normal LVEF and no previous LGE. Strain calculated using either feature tracking or myocardial tagging was predictive of LGE presence and extent with similar efficacy. It should be noted that HARP analyzes mid-myocardial strain while feature tracking analyzes endocardial strain. There is debate about which one is more relevant clinically, but we suspect that the difference is a result of the underlying software differences. Apical LGE was better predicted by ε_cc-tag_, however, this likely is of less clinical importance given that the apex in DMD is relatively spared until late in the disease. It is still unclear what outcome measures are best used for DMD cardiomyopathy. LVEF and LGE have been the standard because clinicians have the most experience with their use and interpretation [11]. It is possible that more advanced methods, such as native T1, ECV, and strain, will supplant LVEF and LGE for assessment of DMD cardiomyopathy severity and prediction of progression, but this study was not designed to determine the optimal CMR biomarkers, only whether non-contrast sequences can predict LGE.

Emphasizing the importance of limiting contrast exposure, a recent study demonstrated no difference in medical management seen with traditional versus conservative contrast administration in DMD patients [41]. Of note, the conservative contrast group still received contrast 84% of the time, suggesting that conservative contrast administration may be just the first step in decreasing cumulative contrast load in DMD. While our institution has been using conservative gadolinium administration for the past 3 years, non-contrast sequences, particularly ε_ls_ and Ɛ_cc_, have potential to help in further reducing contrast administration in this patient population.

Given the substantial mobility challenges and other morbidities, such as contractures and severe scoliosis, seen in DMD patients, reduction in scanner time is an important consideration for patient comfort as well as patient compliance for breath holding in order to obtain the best quality studies. Based on our institutional data, the average total scan time was 39.6 min, with 10 min attributed to post-contrast imaging from sequences that would not have been used if contrast was not administered. Thus, eliminating contrast administration would allow for sub-30 min studies in most cases, which is an important consideration for this patient population in particular, but may also be important in centers were scanner access is limited.

## Limitations

This study is limited by the fact that only 33% (18 of 55 patients) had a change in GSS ≥ 1; a larger change may have improved model performance. Additionally, 70% of patients had LGE severity score of at least 1 on their initial scan. This limited our ability to do subset analysis with sufficient power comparing the severity score 0 group alone with those ≥ 1. As discussed above, native T1 may not be completely linearly related to LGE extent and this may bias the data toward non-significance when there actually is a detectable difference for larger changes in LGE severity. Additionally, the variation in native T1 values is inversely related to flip angle, with lower flip angles having higher standard deviations between measurements [36]. We chose to use MOLLI due to its wide availability, which potentially make our findings more generalizable. However, it is possible that other methods of T1 mapping, such as SASHA, would provide a better prediction of LGE. There were some patients that were excluded because we were not able to obtain IV access in order to administer contrast and a very small minority had to stop early due to poor endurance or discomfort. These patients were not included if they did not complete the protocol; as this was a small minority of patients, we do not believe this led to any bias in the results. It should be noted that any patient with a heart rate > 90 bpm used the high heart rate version of the T1 map. In our data set, 72% of the CMRs were performed in the setting of heart rates > 90 bpm. While this sequence increases the voxel size by decreasing the matrix size, this correction significantly decreases the sensitivity of MOLLI to heart rates at longer T1 times. Our prior work demonstrated no significant effect of HR on T1 times using these parameters [21]. Further investigation with a larger, multicenter cohort may allow for better resolution of detectible differences in native T1 in patients who have small changes in the extent of LGE.

Most of the patients assessed were on steroids or cardiovascular medications. While medications may temper the progression of LGE, which could certainly increase the number of patients with no change in LGE on longitudinal assessment, therapies are unlikely to substantially change the predictive potential of these sequences. Despite these therapies, there does appear to be a relationship with mortality. It is important to assess prediction in a clinically relevant setting, and all of these medications represent standard therapies at most institutions.

Finally, another limitation of this analysis is that the models will require validation secondary to the large number of multiple comparisons.

## Conclusions

In this cohort of DMD boys, models using pre-contrast sequences are predictive of presence and severity of LGE for a particular CMR using both feature tracking and myocardial tagging. For longitudinal assessment for individual patients, ε_cc-tag_ was superior for identification of change in severity by FWHM and ε_ls_ was superior for change in GSS. Although these models were predictive, they were only modestly so, suggesting that non-contrast studies cannot currently replace LGE imaging. Given the critical clinical data provided by LGE, we suggest that DMD subjects should continue to receive gadolinium contrast using a conservative protocol for clinical management. Non-contrast imaging should only be used to estimate LGE when contrast cannot be administered (renal failure or no intravenous access) or if total time must be decreased due to physical limitations.

## Supplementary Information

**Additional file 1:** **Figure S1.** Univariate Prediction Models for LVEF, LVESV_in_, and LVEDV_in_. Models demonstrate modest areas under the curve (AUC) for prediction of presence/absence of LGE for LVEF (A), LVESV_in_ (B) and poor performance for LVEDV_in_ (C). LVEF does well for predicting high grade (GSS ≥ 3) vs low grade (GSS ≤ 2) LGE (D), with less robust performance by LVESVin (E) and LVEDVin (F).

**Additional file 2:** **Figure S2. **Analysis of missing data points in longitudinal cohort. The variable with the highest proportion of missing values was change in basal ɛ_ls_ (A). Combination analysis was performed with blue squares indicating observed data points and red squares indicating missing data (B). This demonstrated the majority of CMRs had all data points (indicated by the bar graph to the right of the matrix), with the next most common being missing only change in basal ɛ_ls_.

**Additional file 3: Table S1.** Models for prediction of presence and severity of LGE (myocardial tagging).

**Additional file 4: Table S2. **Models for prediction of presence/absence LGE and FWHM by segment (myocardial tagging).

**Additional file 5:** **Table S3. **Models for prediction of FWHM and change in FWHM by segment (feature tracking).

**Additional file 6: Table S4. **Models for prediction of change in LGE severity by segment (myocardial tagging).

## Data Availability

The datasets used and/or analyzed during the current study are available from the corresponding author on reasonable request.

## Abbreviations

ε_cc_: Circumferential strain
ε_cc-tag_: Circumferential strain calculated from myocardial tagging
ε_ls_: Longitudinal strain
bSSFP: Balanced steady state free precession
CMR: Cardiovascular magnetic resonance
DMD: Duchenne muscular dystrophy
ECG: Electrocardiogram
ECV: Extracellular volume fraction
FWHM: Full width half maximum
GSS: Global severity score
HARP: Harmonic phase magnetic resonance
LGE: Late gadolinium enhancement
LV: Left ventricle/left ventricular
LVEDV: Left ventricular end-diastolic volume
LVEDVI: Left ventricular end-diastolic volume index
LVEF: Left ventricular ejection fraction
LVESV: Left ventricular end-systolic volume
LVESVI: Left ventricular end-systolic volume index
MOLLI: Modified Look-Locker inversion recovery
PSIR: Phase sensitive inversion recovery
RV: Right ventricle/right ventricular
SASHA: Saturation recovery single-shot acquisition
