# Supplementary figures and images for: Non-contrast cardiovascular magnetic resonance detection of myocardial fibrosis in Duchenne muscular dystrophy
Source: J Cardiovasc Magn Reson. 2021 Apr 29;23:48. doi: 10.1186/s12968-021-00736-1 (PMC8082768; doi:10.1186/s12968-021-00736-1)

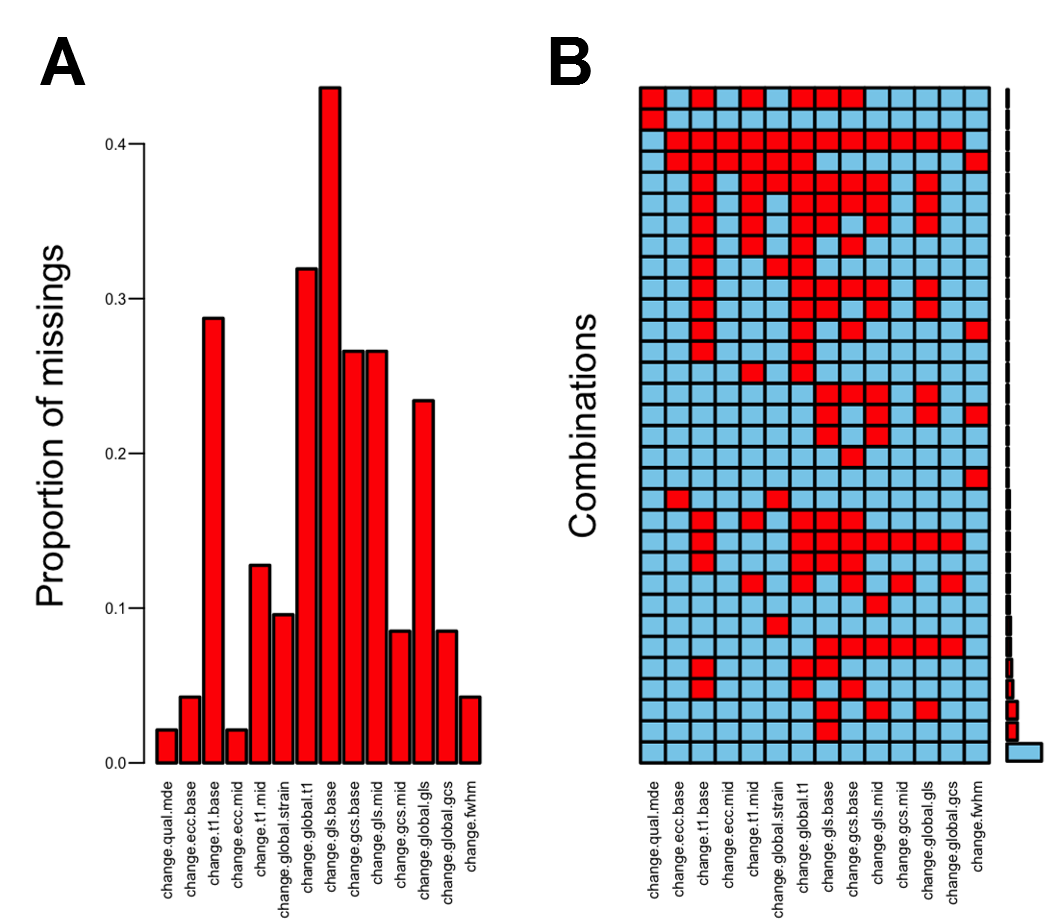

Supplement: Supplementary file 1 — Additional file 1: Figure S1. Univariate Prediction Models for LVEF, LVESVin, and LVEDVin. Models demonstrate modest areas under the curve (AUC) for prediction of presence/absence of LGE for LVEF (A), LVESVin (B) and poor performance for LVEDVin (C). LVEF does well for predicting high grade (GSS ≥ 3) vs low grade (GSS ≤ 2) LGE (D), with less robust performance by LVESVin (E) and LVEDVin (F). [file 12968_2021_736_MOESM1_ESM.tif]

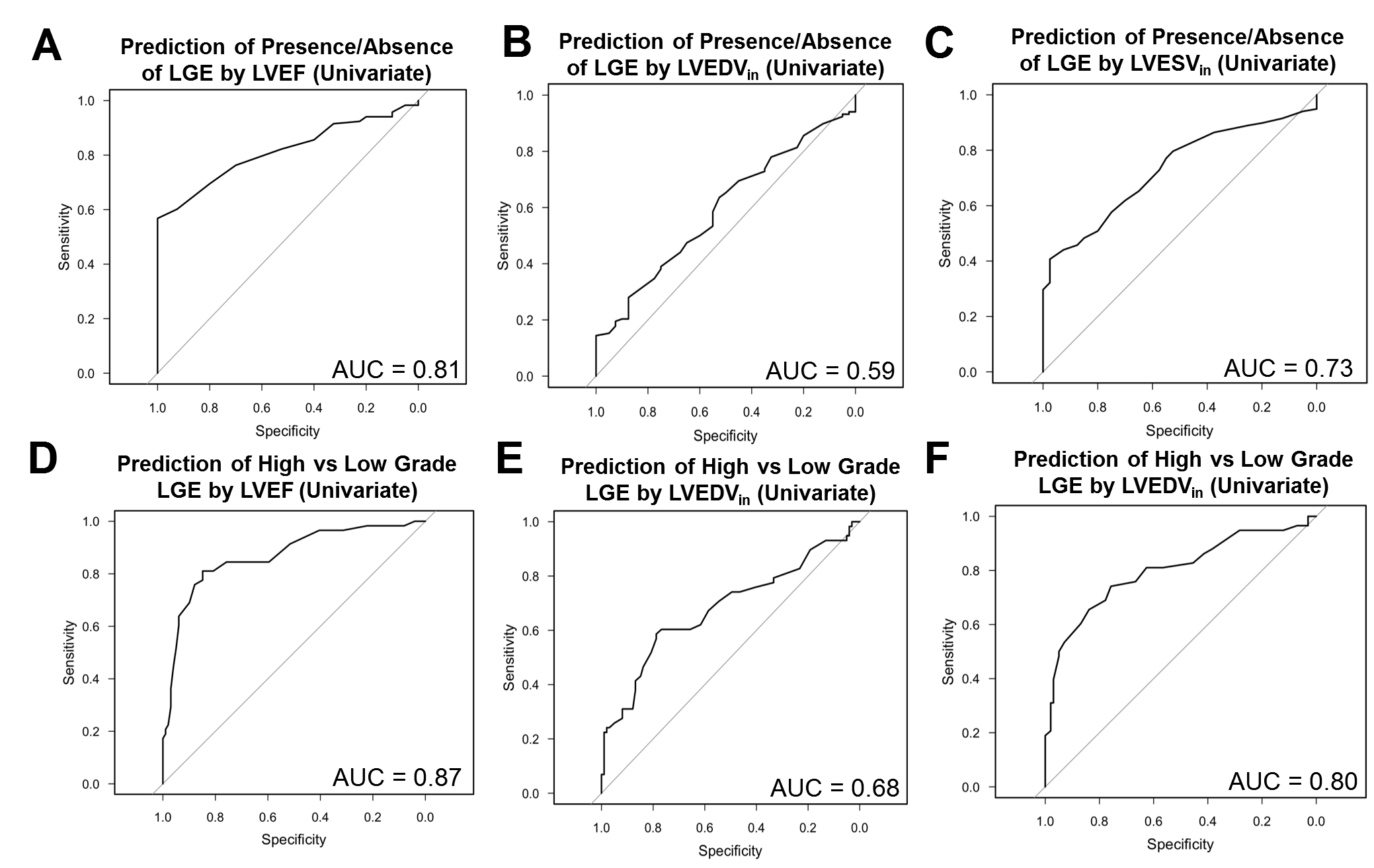

Supplement: Supplementary file 2 — Additional file 2: Figure S2. Analysis of missing data points in longitudinal cohort. The variable with the highest proportion of missing values was change in basal ɛls (A). Combination analysis was performed with blue squares indicating observed data points and red squares indicating missing data (B). This demonstrated the majority of CMRs had all data points (indicated by the bar graph to the right of the matrix), with the next most common being missing only change in basal ɛls. [file 12968_2021_736_MOESM2_ESM.tif]
