# Supplementary material for: Non-contrast cardiovascular magnetic resonance detection of myocardial fibrosis in Duchenne muscular dystrophy
Source: J Cardiovasc Magn Reson. 2021 Apr 29;23:48. doi: 10.1186/s12968-021-00736-1 (PMC8082768; doi:10.1186/s12968-021-00736-1)
Supplement: Supplementary file 3 — Additional file 3: Table S1. Models for prediction of presence and severity of LGE (myocardial tagging). [file 12968_2021_736_MOESM3_ESM.docx]

**Table S1: Models for Prediction of Presence and Severity of LGE (Myocardial Tagging)**

| **Slice** | **Factor** | **Odds Ratio and 95% CI** | | ***p* value** |
| --- | --- | --- | --- | --- |
| **Presence/Absence of LGE** | | | | |
| Base | Native T1 | 1.8 [ 1.1, 3.0] | | ***0.028*** |
| (*n* = 138) | Ԑ_cc-tag_ | 3.0 [1.7, 5.3] | | ***0.0002*** |
| Mid | Native T1 | 1.0[ 0.7, 1.4] | | 0.91 |
| (*n* = 158) | Ԑ_cc-tag_ | 3.4 [1.7, 6.9] | | ***0.0008*** |
| Apex | Native T1 | 1.0 [ 0.5, 2.1] | | 0.94 |
| (*n* = 129) | Ԑ_cc-tag_ | 3.9 [1.3, 5.4] | | ***0.007*** |
| Global | Native T1 | 1.4 [0.8, 2.2] | | 0.22 |
| (*n* = 132) | Ԑ_cc-tag_ | 3.3 [1.7, 6.4] | | ***0.0006*** |
| **Global Severity Score** | | | | |
| Base | Native T1 | 2.0 [ 1.1, 3.7] | ***0.020*** | |
| (*n* = 137) | Ԑ_cc-tag_ | 3.3 [2.0, 5.4] | ***< 0.0001*** | |
| Mid | Native T1 | 1.1 [ 0.8, 1.5] | 0.57 | |
| (*n* = 157) | Ԑ_cc-tag_ | 4.1 [2.5, 6.8] | ***< 0.0001*** | |
| Apex | Native T1 | 0.7 [ 0.5, 1.2] | 0.24 | |
| (*n* = 128) | Ԑ_cc-tag_ | 2.6 [1.6, 4.2] | ***0.0002*** | |
| Global | Native T1 | 1.6 [ 1.0, 2.5] | ***0.052*** | |
| (*n* = 131) | Ԑ_cc-tag_ | 2.6 [2.2, 6.4] | ***< 0.0001*** | |
| **FWHM** | | | | |
| Base | Native T1 | 1.4 [0.7, 3.0] | 0.38 | |
| (*n* = 58) | Ԑ_cc-tag_ | 2.7 [1.5, 4.8] | ***0.001*** | |
| Mid | Native T1 | 1.0 [ 0.7, 1.3] | 0.76 | |
| (*n* = 156) | Ԑ_cc-tag_ | 2.4 [1.6, 3.7] | ***< 0.0001*** | |
| Apex | Native T1 | 0.9 [ 0.6, 1.3] | 0.47 | |
| (*n* = 126) | Ԑ_cc-tag_ | 1.4 [1.0, 1.9] | ***0.047*** | |
| Global | Native T1 | 1.3 [ 0.8, 2.0] | 0.30 | |
| (*n* = 130) | Ԑ_cc-tag_ | 1.9 [1.3, 2.9] | ***0.001*** | |
