# Supplementary material for: Non-contrast cardiovascular magnetic resonance detection of myocardial fibrosis in Duchenne muscular dystrophy
Source: J Cardiovasc Magn Reson. 2021 Apr 29;23:48. doi: 10.1186/s12968-021-00736-1 (PMC8082768; doi:10.1186/s12968-021-00736-1)
Supplement: Supplementary file 4 — Additional file 4: Table S2. Models for prediction of presence/absence LGE and FWHM by segment (myocardial tagging). [file 12968_2021_736_MOESM4_ESM.docx]

**Table S2: Models for Prediction of Presence/Absence LGE and FWHM by Segment (Myocardial Tagging)**

|  |  | **LGE Presence/Absence** |  | **FWHM** |  |
| --- | --- | --- | --- | --- | --- |
| **Segment** | **Factor** | **Odds Ratio and 95% CI** | ***p* value** | **Odds Ratio and 95% CI** | ***p* value** |
| Basal Anterior | Native T1 | 0.9 [0.6, 1.5] | 0.80 | 1.1 [0.8, 1.7] | 0.50 |
|  | Ԑ_cc-tag_ | 0.9 [0.4, 1.8] | 0.74 | 1.0 [0.5, 1.8] | 0.90 |
| Basal Anteroseptal | Native T1 | 0.8 [0.4, 1.3] | 0.33 | 1.2 [0.7, 1.9] | 0.53 |
|  | Ԑ_cc-tag_ | 1.1 [0.7, 1.9] | 0.66 | 0.8 [0.5, 1.2] | 0.24 |
| Basal Inferoseptal | Native T1 | 1.3 [0.7, 2.3] | 0.42 | 1.1 [0.9, 1.3] | 0.62 |
|  | Ԑ_cc-tag_ | 1.7 [1.0, 2.8] | 0.06 | 2.5 [1.7, 3.6] | ***< 0.001*** |
| Basal Inferior | Native T1 | 1.6 [0.8, 3.1] | 0.21 | 1.0 [0.7, 1.4] | 0.97 |
|  | Ԑ_cc-tag_ | 1.5 [1.0, 2.4] | 0.08 | 1.6 [1.1, 2.3] | ***0.02*** |
| Basal Inferolateral | Native T1 | 2.9 [1.3, 6.8] | ***0.014*** | 1.4 [1.0, 1.9] | 0.07 |
|  | Ԑ_cc-tag_ | 3.2 [1.5, 6.8] | ***0.003*** | 1.7 [1.0, 2.8] | ***0.041*** |
| Basal Anterolateral | Native T1 | 1.5 [1.1, 2.2] | ***0.045*** | 1.2 [0.9, 1.6] | 0.16 |
|  | Ԑ_cc-tag_ | 3.2 [1.5, 7.1] | ***0.004*** | 2.5 [1.5, 4.1] | ***< 0.001*** |
| Mid Anterior | Native T1 | 0.5 [0.3, 0.9] | ***0.019*** | 1.0 [0.7, 1.4] | 0.92 |
|  | Ԑ_cc-tag_ | 1.2 [0.6, 2.3] | 0.67 | 1.4 [0.8, 2.5] | 0.20 |
| Mid Anteroseptal | Native T1 | 1.0 [1.0, 1.1] | ***0.002*** | 1.0 [0.9, 1.0] | 0.66 |
|  | Ԑ_cc-tag_ | 1.3 [0.7, 2.5] | 0.39 | 1.1 [0.6 2.2] | 0.77 |
| Mid Inferoseptal | Native T1 | 1.1 [0.8, 1.6] | 0.62 | 1.0 [0.9, 1.2] | 0.51 |
|  | Ԑ_cc-tag_ | 1.7 [1.1, 2.7] | ***0.03*** | 2.0 [1.3, 3.1] | ***0.003*** |
| Mid Inferior | Native T1 | 0.5 [0.2, 1.4] | 0.19 | 0.4 [0.1, 1.4] | 0.16 |
|  | Ԑ_cc-tag_ | 4.1 [1.6, 10.6] | ***0.004*** | 3.1 [1.8, 5.4] | ***< 0.001*** |
| Mid Inferolateral | Native T1 | 1.0 [0.6, 1.5] | 0.88 | 0.9 [0.7, 1.1] | 0.44 |
|  | Ԑ_cc-tag_ | 6.0 [2.3, 15.2] | ***< 0.001*** | 2.3 [1.4, 3.9] | ***0.001*** |
| Mid Anterolateral | Native T1 | 0.8 [0.4, 1.4] | 0.37 | 0.8 [0.6, 1.3] | 0.42 |
|  | Ԑ_cc-tag_ | 3.0[1.7, 5.1] | ***< 0.001*** | 1.9 [1.3, 2.9] | ***0.001*** |
