# Supplementary material for: Non-contrast cardiovascular magnetic resonance detection of myocardial fibrosis in Duchenne muscular dystrophy
Source: J Cardiovasc Magn Reson. 2021 Apr 29;23:48. doi: 10.1186/s12968-021-00736-1 (PMC8082768; doi:10.1186/s12968-021-00736-1)
Supplement: Supplementary file 5 — Additional file 5: Table S3. Models for prediction of FWHM and change in FWHM by segment (feature tracking). [file 12968_2021_736_MOESM5_ESM.docx]

**Table S3: Models for Prediction of FWHM and Change in FWHM by Segment (Feature Tracking)**

|  |  | **FWHM** |  | | **Change in FWHM** |  | | |  |  |
| --- | --- | --- | --- | --- | --- | --- | --- | --- | --- | --- |
| **Segment** | **Factor** | **Odds Ratio and 95% CI** | | ***p* value** | **Odds Ratio and 95% CI** | ***p* value** |  |  | | |
| Basal Anterior | Native T1 | 1.0 [0.7, 1.5] | | 0.85 | 1.2 [0.8, 1.8] | 0.47 |  |  | | |
|  | Ԑ_cc_ | 0.9 [0.4, 1.8] | | 0.75 | 2.1 [0.5, 8.2] | 0.30 |  |  | | |
|  | Ԑ_ls_ | 1.1 [0.6, 2.1] | | 0.84 | 0.4 [0.2, 0.9] | ***0.022*** |  |  | | |
| Basal Anteroseptal | Native T1 | 1.0 [0.5, 2.0] | | 1.0 | 0.4 [0.2, 0.9] | **0.*036*** |  |  | | |
|  | Ԑ_cc_ | 0.8 [0.3, 2.0] | | 0.69 | 0.6 [0.2, 1.5] | 0.25 |  |  | | |
|  | Ԑ_ls_ | 1.4 [0.7, 2.7] | | 0.29 | 2.5 [1.4, 4.6] | ***0.002*** |  |  | | |
| Basal Inferoseptal | Native T1 | 0.9 [0.9, 1.1] | | 0.28 | 0.9 [0.7, 1.1] | 0.36 |  |  | | |
|  | Ԑ_cc_ | 1.0 [0.6, 1.7] | | 0.92 | 2.9 [1.1, 8.0] | ***0.036*** |  |  | | |
|  | Ԑ_ls_ | 1.1 [0.4, 3.2] | | 0.86 | 1.3 [0.4, 3.7] | 0.68 |  |  | | |
| Basal Inferior | Native T1 | 1.2 [0.9, 1.8] | | 0.23 | 2.3 [1.4, 1.8] | ***0.001*** |  |  | | |
|  | Ԑ_cc_ | 1.6 [1.0, 2.5] | | 0.069 | 1.3 [0.7, 6.2] | 0.46 |  |  | | |
|  | Ԑ_ls_ | 1.8 [1.1, 2.8 | | ***0.013*** | 1.1 [0.4, 2.6] | 0.89 |  |  | | |
| Basal Inferolateral | Native T1 | 1.5 [1.0, 2.1] | | ***0.036*** | 0.5 [0.1, 2.0] | 0.34 |  |  | | |
|  | Ԑ_cc_ | 1.4 [0.8, 2.4] | | 0.25 | 2.1 [0.7, 6.2] | 0.19 |  |  | | |
|  | Ԑ_ls_ | 1.1 [0.6, 1.8] | | 0.87 | 1.0 [0.1, 8.3] | 0.98 |  |  | | |
| Basal Anterolateral | Native T1 | 0.9 [0.7, 1.1] | | 0.42 | 1.0 [0.3, 3.3] | 0.97 |  |  | | |
|  | Ԑ_cc_ | 2.4 [1.4, 4.2] | | ***0.001*** | 0.8 [0.4, 1.5] | 0.45 |  |  | | |
|  | Ԑ_ls_ | 0.9 [0.5, 1.6] | | 0.71 | 1.9 [0.6, 6.2] | 0.27 |  |  | | |
| Mid Anterior | Native T1 | 0.8 [0.5, 1.1] | | 0.11 | 0.8 [0.5, 1.4] | 0.46 |  |  | | |
|  | Ԑ_cc_ | 1.2 [0.8, 1.7] | | 0.52 | 1.0 [0.4, 2.2] | 0.96 |  |  | | |
|  | Ԑ_ls_ | 1.1 [0.7, 1.7] | | 0.65 | 1.1 [0.6, 2.3] | 0.72 |  |  | | |
| Mid Anteroseptal | Native T1 | 1.0 [1.0, 1.0] | | ***< 0.001*** | 1.0 [0.9, 1.0] | 0.47 |  |  | | |
|  | Ԑ_cc_ | 1.0 [0.6 1.7] | | 0.96 | 0.8 [0.4 1.5] | 0.45 |  |  | | |
|  | Ԑ_ls_ | 1.1 [0.8, 1.7] | | 0.55 | 0.5 [0.2, 1.3] | 0.15 |  |  | | |
| Mid Inferoseptal | Native T1 | 0.6 [0.4, 1.1] | | 0.10 | 0.8 [0.4, 1.7] | 0.51 |  |  | | |
|  | Ԑ_cc_ | 1.4 [0.9, 2.2] | | 0.18 | 1.9 [0.6, 5.6] | 0.27 |  |  | | |
|  | Ԑ_ls_ | 1.6 [1.0, 2.6] | | ***0.05*** | 1.2 [0.6, 2.8] | 0.60 |  |  | | |
| Mid Inferior | Native T1 | 1.3 [0.9, 1.6] | | 0.15 | 1.2 [0.7, 1.8] | 0.54 |  |  | | |
|  | Ԑ_cc_ | 1.3 [0.8, 2.0] | | 0.24 | 0.9 [0.1, 6.1] | 0.93 |  |  | | |
|  | Ԑ_ls_ | 1.1 [0.7, 1.8] | | 0.59 | 1.0 [0.7, 1.4] | 0.92 |  |  | | |
| Mid Inferolateral | Native T1 | 1.2 [0.9, 1.6] | | 0.17 | 0.8 [0.4, 1.7] | 0.53 |  |  | | |
|  | Ԑ_cc_ | 1.1 [0.9, 1.3] | | 0.40 | 0.8 [0.2, 3.4] | 0.80 |  |  | | |
|  | Ԑ_ls_ | 1.3[0.8, 2.1] | | 0.32 | 0.7[0.3, 1.4] | 0.27 |  |  | | |
| Mid Anterolateral | Native T1 | 0.6 [0.3, 1.1] | | 0.10 | 0.7 [0.3, 1.2] | 0.18 |  |  | | |
|  | Ԑ_cc_ | 0.8 [0.4, 1.8] | | 0.63 | 0.8 [0.6, 1.0] | ***0.039*** |  |  | | |
|  | Ԑ_ls_ | 2.0 [0.9, 4.2] | | 0.08 | 1.1 [0.6, 1.7] | 0.86 |  |  | | |
