# Supplementary material for: Non-contrast cardiovascular magnetic resonance detection of myocardial fibrosis in Duchenne muscular dystrophy
Source: J Cardiovasc Magn Reson. 2021 Apr 29;23:48. doi: 10.1186/s12968-021-00736-1 (PMC8082768; doi:10.1186/s12968-021-00736-1)
Supplement: Supplementary file 6 — Additional file 6: Table S4. Models for prediction of change in LGE severity by segment (myocardial tagging). [file 12968_2021_736_MOESM6_ESM.docx]

**Table S4: Models for Prediction of Change in LGE Severity by Segment (Myocardial Tagging)**

|  |  | **Change in Severity Score** |  | **Change in FWHM** |  |
| --- | --- | --- | --- | --- | --- |
| **Segment** | **Factor** | **Odds Ratio and 95% CI** | ***p* value** | **Odds Ratio and 95% CI** | ***p* value** |
| Basal Anterior | ∆Native T1 | 0.6 [0.2, 2.2] | 0.43 | 1.4 [1.0, 2.1] | 0.08 |
|  | ∆Ԑ_cc-tag_ | 0.6 [0.4, 0.8] | ***0.001*** | 0.4 [0.1, 1.1] | 0.08 |
| Basal Anteroseptal | ∆Native T1 | 0.2 [0.1, 0.7] | ***0.008*** | 2.1 [0.8, 5.4] | 0.13 |
|  | ∆Ԑ_cc-tag_ | 0.1 [0, 0.4] | ***0.002*** | 0.8 [0.4, 1.5] | 0.48 |
| Basal Inferoseptal | ∆Native T1 | 0.5 [1.0, 2.1] | ***0.036*** | 0.9 [0.7, 1.1] | 0.90 |
|  | ∆Ԑ_cc-tag_ | 0 [0, 0.4] | ***0.019*** | 2.9 [1.1, 8.0] | 0.48 |
| Basal Inferior | ∆Native T1 | 1.0 [0.8, 1.2] | 0.64 | 2.0 [1.3, 3.0] | ***0.002*** |
|  | ∆Ԑ_cc-tag_ | 0.4 [0.2, 1.1] | 0.08 | 0.9 [0.3, 2.9] | 0.91 |
| Basal Inferolateral | ∆Native T1 | 0.3 [0.1, 1.1] | 0.07 | 2.4 [0.8, 7.1] | 0.12 |
|  | ∆Ԑ_cc-tag_ | 3.4 [1.5, 7.7] | ***0.003*** | 0.8 [0.2, 3.1] | 0.74 |
| Basal Anterolateral | ∆Native T1 | 0.4 [0.2, 0.6] | **< 0.001** | 1.6 [1.1, 2.4] | ***0.023*** |
|  | ∆Ԑ_cc-tag_ | 1.1 [0.5, 2.8] | 0.77 | 0.7 [0.3, 1.7] | 0.38 |
| Mid Anterior | ∆Native T1 | 1.0 [0.9, 1.1] | 0.99 | 1.2 [0.7, 2.2] | 0.53 |
|  | ∆Ԑ_cc-tag_ | 0.2 [0.1, 0.6] | ***0.002*** | 1.6 [0.7, 3.8] | 0.29 |
| Mid Anteroseptal | ∆Native T1 | 1.0 [1.0, 1.0] | ***< 0.001*** | 1.0 [0.9, 1.0] | ***< 0.001*** |
|  | ∆Ԑ_cc-tag_ | 0.4 [0.1, 1.4] | 0.15 | 0.7 [0.3 2.0] | 0.52 |
| Mid Inferoseptal | ∆Native T1 | 0.9 [0.7, 1.2] | 0.52 | 1.1 [1.0, 1.1] | ***0.05*** |
|  | ∆Ԑ_cc-tag_ | 0.7 [0.4, 1.1] | 0.08 | 1.0 [0.4, 2.5] | 0.99 |
| Mid Inferior | ∆Native T1 | 0.2 [0, 1.0] | ***0.04*** | 1.2 [0.7, 2.2] | 0.50 |
|  | ∆Ԑ_cc-tag_ | 1.0 [0.3, 3.0] | 0.99 | 0.9 [0.3, 2.3] | 0.82 |
| Mid Inferolateral | ∆Native T1 | 0.1 [0.1, 0.3] | ***< 0.001*** | 1.4 [0.7, 2.7] | 0.35 |
|  | ∆Ԑ_cc-tag_ | 6.6 [1.5, 28.2] | ***0.011*** | 0.4 [0.2, 0.9] | ***0.028*** |
| Mid Anterolateral | ∆Native T1 | 0.1 [0, 0.6] | ***0.010*** | 1.2 [0.7, 1.9] | 0.51 |
|  | ∆Ԑ_cc-tag_ | 0.5 [0.2, 1.1] | 0.10 | 0.7 [0.3, 1.7] | 0.46 |
